# Supplementary material for: The anticipation of events in time
Source: Nat Commun. 2019 Dec 20;10:5802. doi: 10.1038/s41467-019-13849-0 (PMC6925136; doi:10.1038/s41467-019-13849-0)
Supplement: Supplementary file 1 — Supplementary Information [file 41467_2019_13849_MOESM1_ESM.pdf]

Supplementary Information

The anticipation of events in time

Grabenhorst et al.

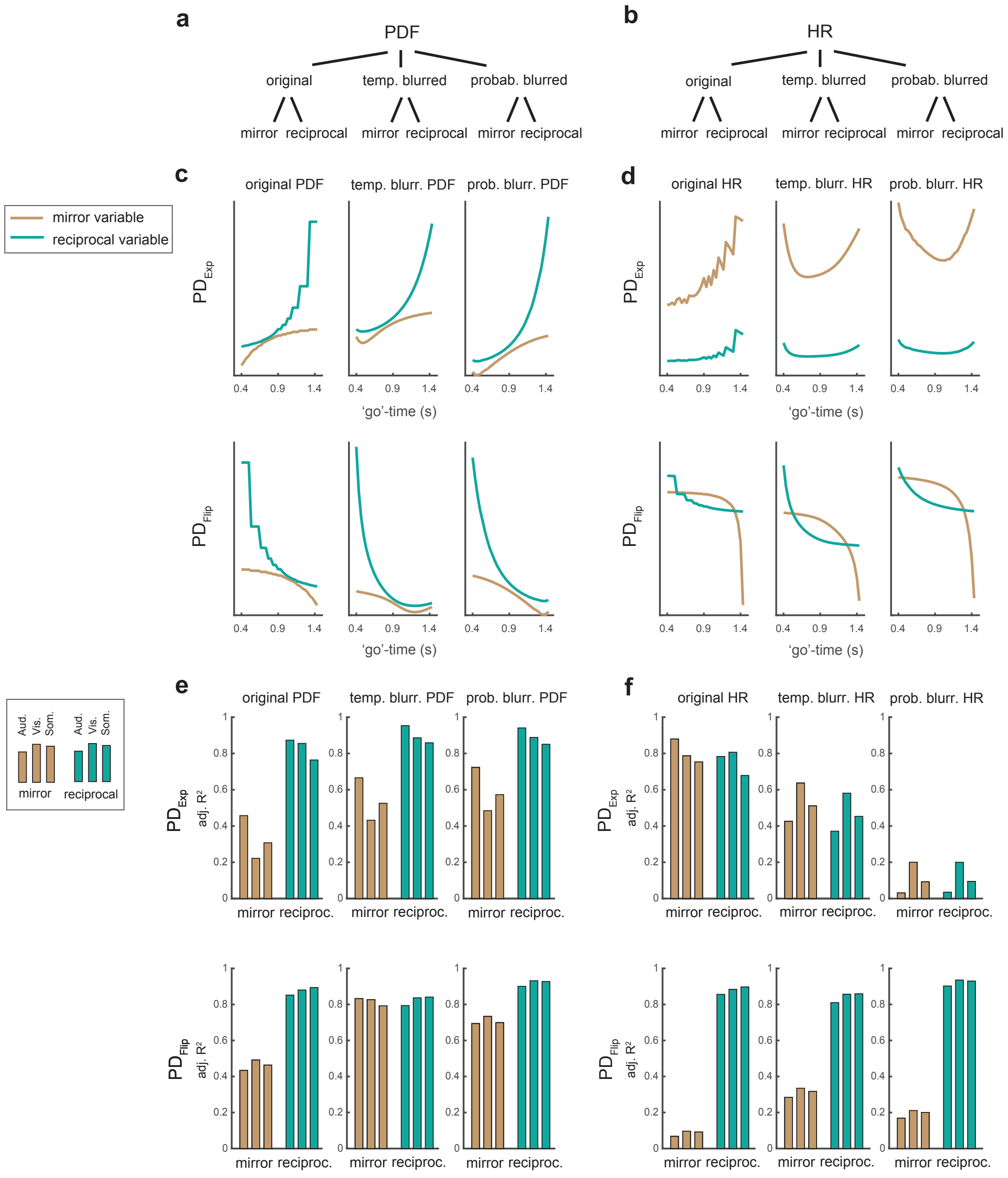

Supplementary Figure 1. Models of reaction time with respect to probability over time. **a** and **b** Schematic illustrating the relationship between a presented PDF or its HR and the derived explanatory variables used to model RT. **c** Exponential and flipped exponential PDFs in original, temporally and probabilistically blurred versions. The original presented 'go' time functions and their blurred versions are shown to highlight the effect of the convolution with a Gaussian distribution whose standard deviation scales either with 'go' time (temporal blurring) or it scales according to the PDF of event occurrence (probabilistic blurring). In case of the PDFs the blurring changes the monotonically decreasing (PD_{Exp}) and increasing (PD_{Flip}) shapes to biphasic curves. **d** In the HRs, the blurring had a stronger effect in the PD_{Exp} condition than in the PD_{Flip} condition. Based on the assumption that RT should be smallest where probability

– or hazard – is highest, an inverse relationship between RT and model was considered. Before fitting the models to the data, all explanatory variables were either mirrored around their mean, which is a linear transformation, or they were transformed into their reciprocal versions, which is a nonlinear transformation. **e, f** Each of the explanatory variables was fitted to RT of the respective condition (PD_{Exp} or PD_{Flip}) in all sensory conditions. Each bar represents the adjusted R^2 value of a single group-level fit.

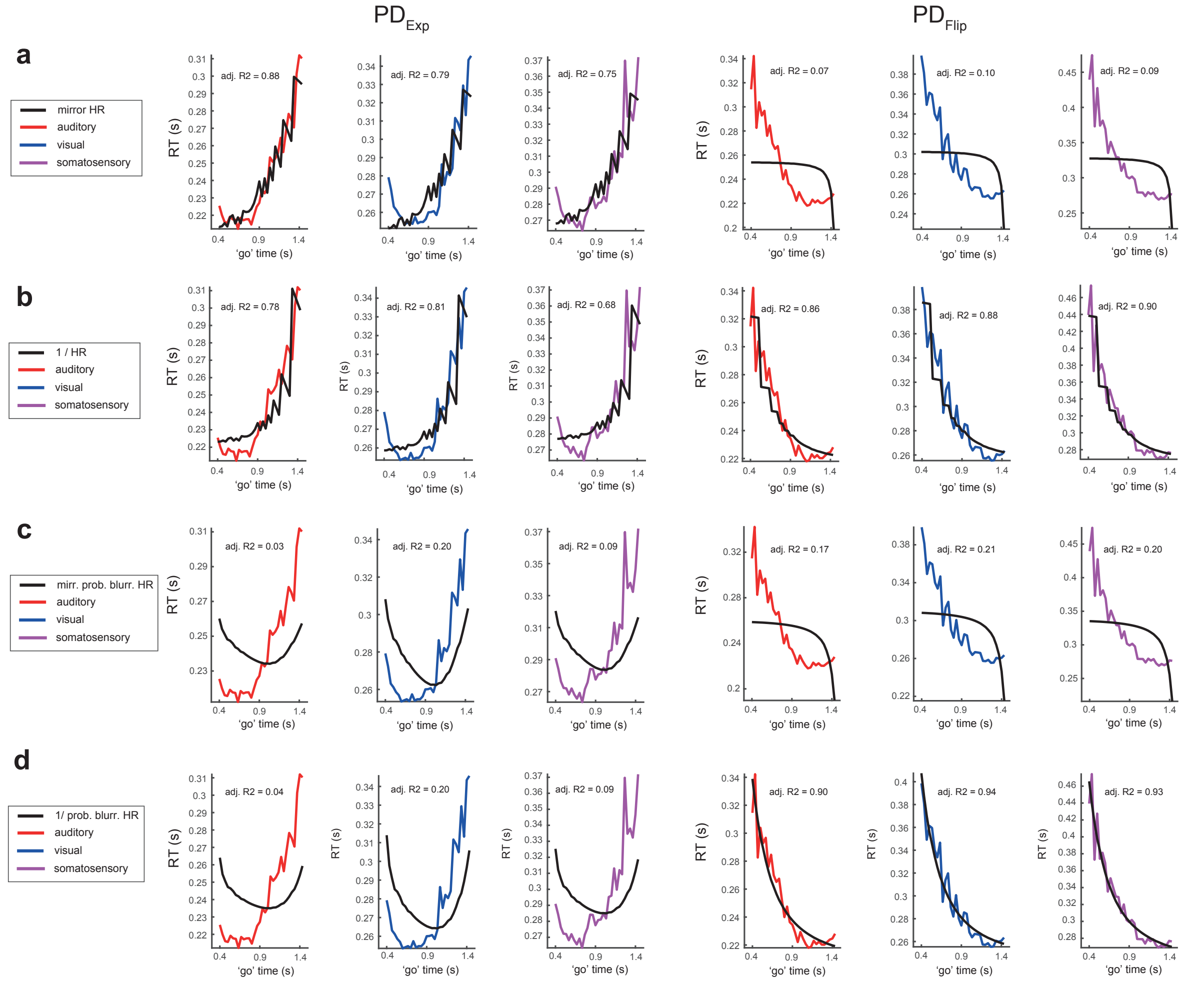

Supplementary Figure 2. Models of RT based on the hazard rate (HR) of 'go' times. **a** The normal HR does not capture the data well in the PD_{Flip} condition. **b** The reciprocal of the HR fits the data better than the mirror one. In the PD_{Exp} condition the model does not capture RTs at shorter 'go' times. Although in the PD_{Flip} condition the model fits the data better and thus gives a relatively high adjusted R^2, the stepped character of the explanatory variable is not reflected in the data. **c** The predictions of the model does not match the data in either the PD_{Exp} condition, or the PD_{Flip} condition. **d** The probabilistically-blurred HR does not capture the pattern of RT in the PD_{Exp} condition. Although in the PD_{Flip} condition the model clearly fits the data better and thus gives a high adjusted R^2, a convincing model needs to capture the RT data in both probabilistic conditions.

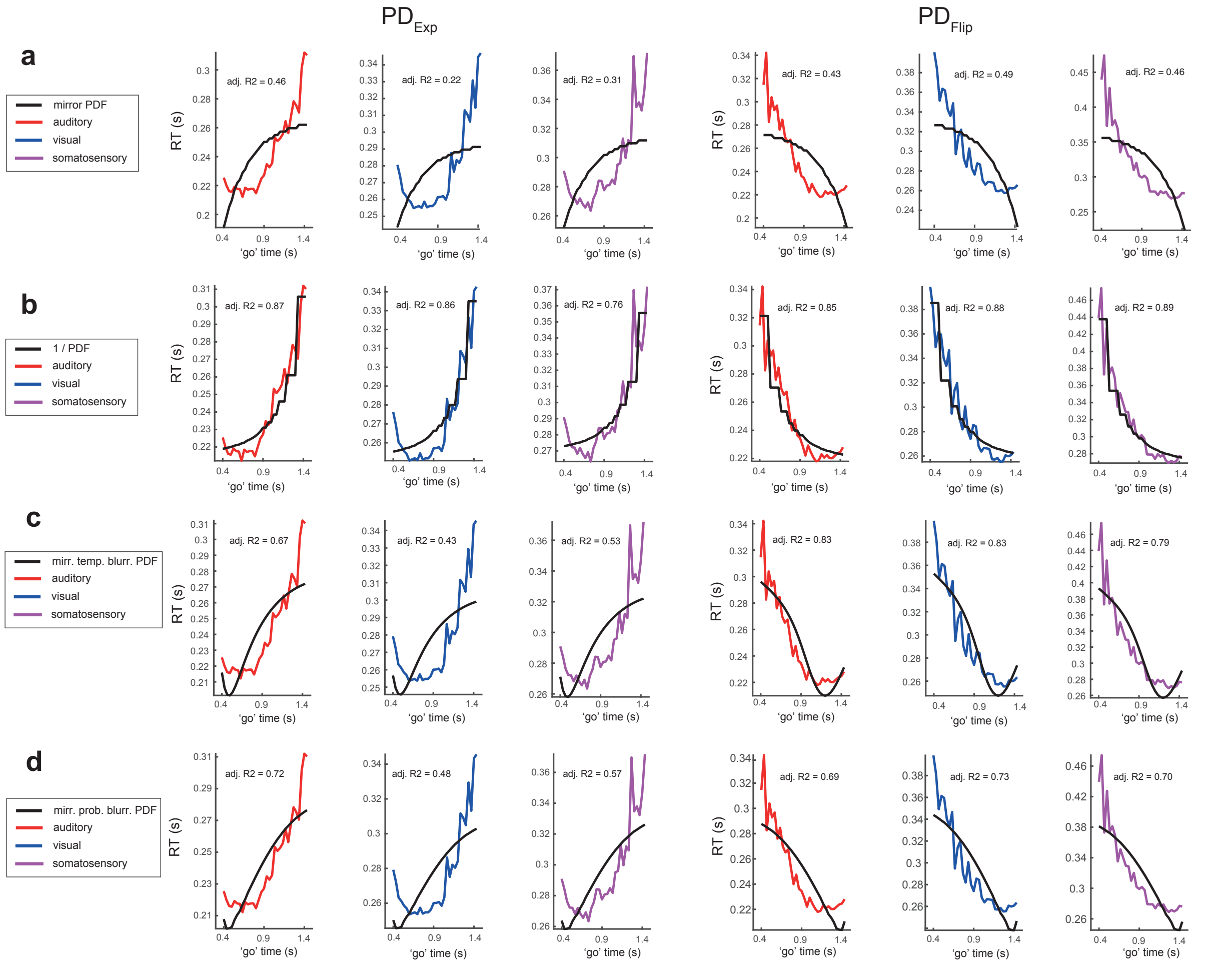

Supplementary Figure 3. Models of RT based on the probability density function (PDF) of 'go' times. **a** The mirrored original PDF (linearly transformed) does not fit the data in either the PD_{Exp} condition or in the PD_{Flip} condition. **b** The original PDF captures the behavior of the RT data well in both the PD_{Exp} condition and the PD_{Flip} condition, as evidenced by the high adjusted R^2. However, the original PDF contains step-discontinuities not evident in the data. **c** The mirrored, temporally-blurred PDF does not fit the data in either the PD_{Exp} condition or in the PD_{Flip} condition. **d** The mirrored probabilistically-blurred PDF does not fit the data well in either the PD_{Exp} condition or in the PD_{Flip} condition.

Supplementary Note 1

Split-data analyses and single-subject analyses. Several control analyses support the validity of the key findings (Figs. 3 and 4).

Comparison of early and late trials within experimental conditions. Each distribution (exponential and flipped exponential) was presented in pairs of two consecutive blocks of trials. To investigate whether RT behavior, as well as corresponding models, was different between early and late parts of these pairs of blocks, RT curves were computed separately from early and late trials. Namely, RT curves were computed only from the first 100 trials of a pair of blocks (early period) and from the last 100 trials from a pair of blocks (late period) within both days 1 and 2. The resulting RT curves were very similar, indicating (i) fast adaptation to the distribution and (ii) behavioral stability within each condition. The mirrored, temporally-blurred HR model (Supplementary Fig. 4) and the reciprocal, probabilistically-blurred PDF model (Supplementary Fig. 5) were fit to RT curves from early and late periods using the linear model described earlier. Importantly, the fitted probabilistically-blurred, reciprocal PDF (Supplementary Fig. 5, black fit lines) again outperformed the canonical temporally-blurred, mirrored HR (Supplementary Fig. 4, black fit lines) as a model of RT in both early and late periods .

Cross-validation of model fits between days 1 and 2. The stability of the model fits was examined by cross-validation. Both PDF-based and HR-based models were fitted to RT from day 1 and were then used to predict the RT from day 2. To do so, the models were then fitted to data from day 2 allowing only for a free offset parameter, b, to account for potential differences in overall RT between days (Supplementary Fig. 6a and b). The scaling parameter was fixed at $\mathrm{a} = 1$ in the linear fitting formula: $\mathrm{f(x)} = \mathrm{ax} + \mathrm{b}$. The PDF-based model from day 1 provided a good fit to the data from day 2 (Supplementary Fig. 6 a and b) while the HR model did not fit the data well (Supplementary Fig. 6 c and d).

Potential effects of the sequence of PDF types. A third control investigated whether the sequence of event PDF *types* affected the RT curves . For half of the participants, the presented sequence was PD_{Exp} – PD_{Flip}, and for the other half PD_{Flip} – PD_{Exp}. The RT curves were calculated for these two groups (Supplementary Fig. 7). To allow for better visual comparison, the RT curves were vertically aligned with respect to the 'go' time with the highest probability (0.4 s in PD_{Exp} and 1.4 s in PD_{Flip}) by substracting the PD_{Exp} RT curve from the PD_{Flip} RT curve. We found that RT curves were highly similar in both groups, indicating no effect of the sequence order.

Single-subjects fits with a fixed Weber fraction for all subjects. We next investigated whether the group-level findings also hold at the single-subject level. Within participants mean RT was calculated for each 'go' time resulting in RT curves. The HR-based and the PDF-based models were fitted to single-subject RT curves to assess the models' potential to capture these data. For all single-subject fit plots displayed in Supplementary Figs. 8 to 11, a fixed Weber fraction of $\varphi = 0.21$ was used. The PDF-based model outperformed the HR-based model in fits to single-subject RT curves qualitatively (Supplementary Fig. 8 and 9) as well as by evaluation based on adjusted R^2 (Supplementary Fig. 10 and 11).

Single-subjects fits with a subject-specific Weber fraction. The potential impact of individual subjects' uncertainty in time estimation on the group-level modeling was examined. Instead of using a fixed Weber fraction of $\varphi = 0.21$ across all subjects (as was done for the group-level fits) to represent the dynamics of uncertainty in time estimation (Gaussian blurring kernel), an individual Weber fraction was estimated for each subject.

The single-subjects' RT curves were fitted with the mirrored, temporally-blurred HR and with the reciprocal, probabilistically-blurred PDF which were each constructed for a range of $\varphi = [0.1{:}\,0.01{:}\,0.3]$. This range of φ covered the range reported in a large body of previous research[1]. For each subject, the adjusted R^2 was calculated for each of the six experimental conditions and adjusted R^2 was rescaled to range between 0 and 1 across experimental conditions. For each subject the value of φ that resulted in the least variance in rescaled adjusted R^2 across the six experimental conditions (audition, vision, somatosensation; PD_{Exp} and PD_{Flip}) was selected for both the HR-based and the PDF-based models. The models were then fitted again to the single-subject RT curves using this subject-specific value of φ. In each of the six experimental conditions, this resulted in 24 fits for the HR-based model and 24 fits for the PDF-based model. The mean predicted RT by the *HR-based model* was then calculated within each 'go' time across all subjects and plotted against the actual group-level RT curves (Supplementary Fig. 12c and d). Adjusted R^2 was calculated to assess the goodness-of-fit. The same was done for the *PDF-based model* (Supplementary Fig. 12a and b). These fits, based on subject-specific Weber fractions, yielded highly similar results to those derived from a fixed Weber fraction ($\varphi = 0.21$, see Methods) for both the HR-based (Fig. 3a and b) and PDF-based models (Fig. 4c and d), supporting the inferences from the initial modeling strategy with a fixed Weber fraction for the group-level fits.

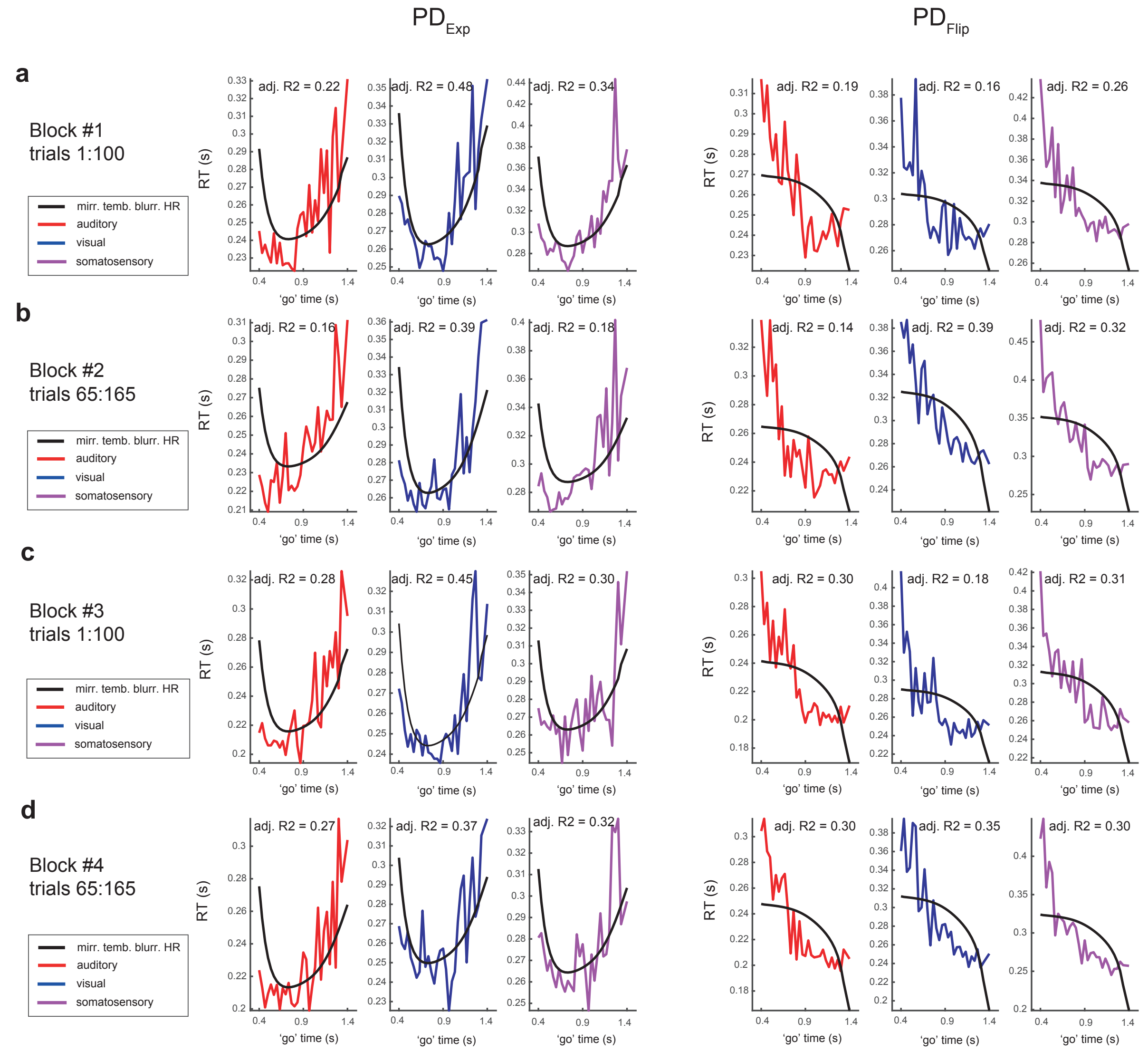

Supplementary Figure 4. Comparison of hazard rate (HR) model fit between early trials in a condition and late trials in a condition. To investigate whether RT behavior changes within a condition over the course of two experimental blocks, RTs from the first 100 trials of a pair of blocks and from the last 100 trials from a pair of blocks were selected in both day 1 (block #1, see **a,** and block #2, see **b**) and day 2 (block #3, see **c**, and block #4, see **d**). In each condition (sensory modality and event distribution) the RT curves are very similar across the selected data subsets, indicating stability in RT behavior. The mirrored, temporally-blurred HR model was fit to RT from each subset and could not acount for the data.

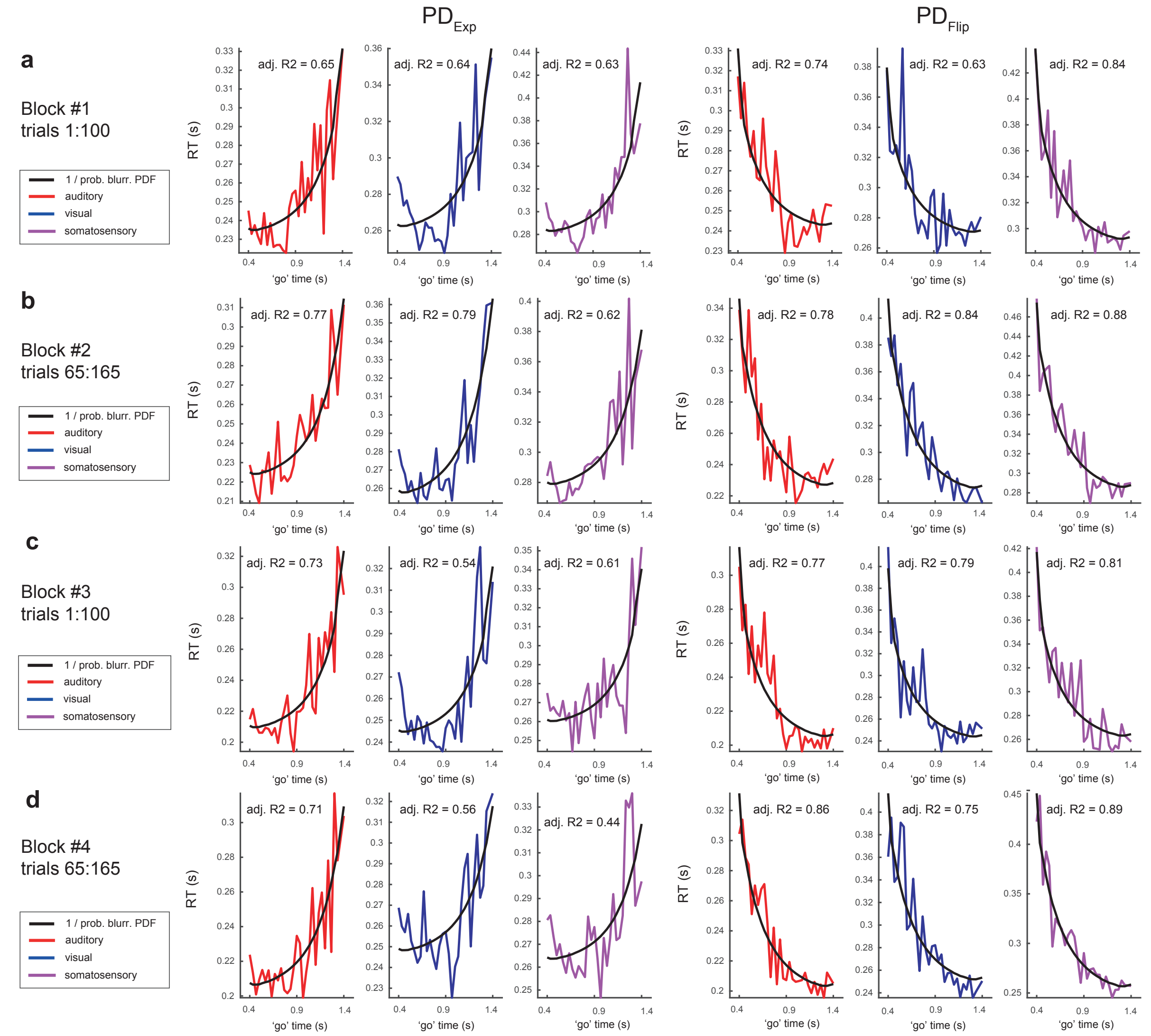

Supplementary Figure 5. Comparison of PDF-based model fit between early trials in a condition and late trials in a condition. To investigate whether RT behavior changes within a condition over the course of two experimental blocks, RTs from the first 100 trials of a pair of blocks and from the last 100 trials from a pair of blocks were selected in both day 1 (block #1, see **a,** and block #2, see **b**) and day 2 (block #3, see **c**, and block #4, see **d**). The reciprocal, probabilistically-blurred PDF model fit to RT from each subset captured the data better than the prominent HR-based model (Suppl. Fig. 4).

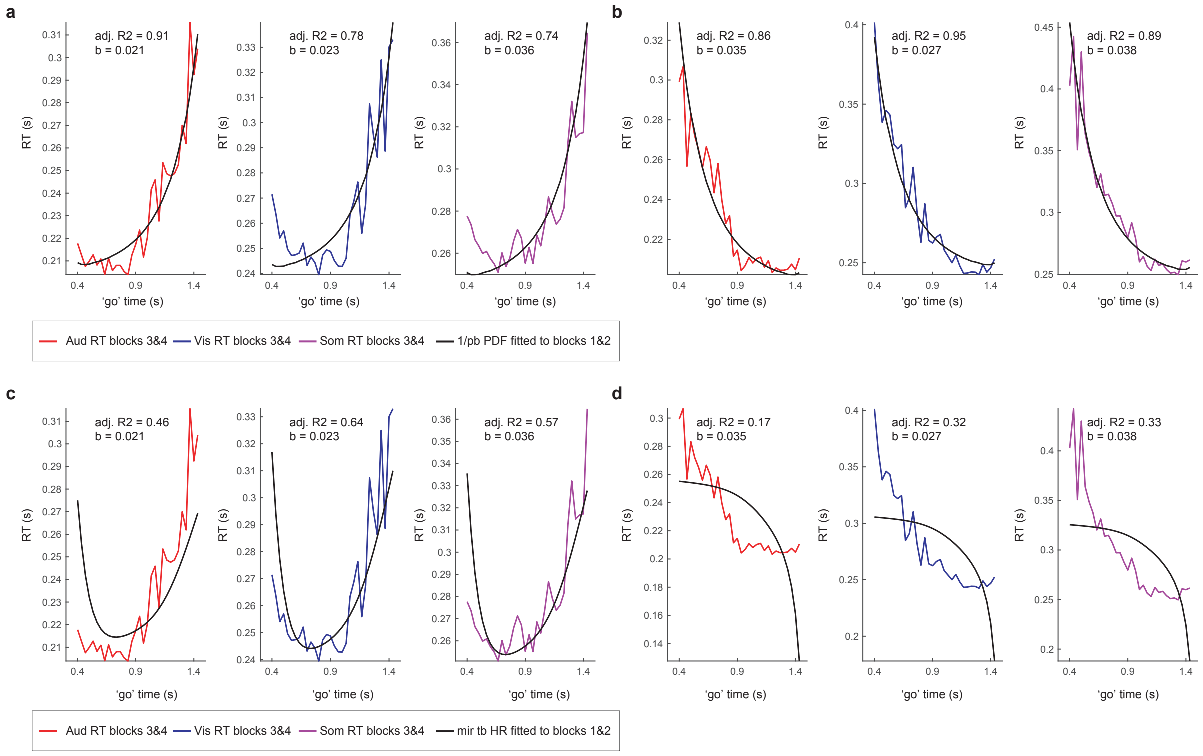

Supplementary Figure 6. Cross-validation of model fits between days 1 and 2. Reciprocal probabilistically-blurred PDF model fitted to data from the first half of the experiment fitted data from the last half of the experiment, indicating stability of behavior and model fit in both PD_{Exp}, see **a**, and PD_{Flip} conditions, see **b**. **c, d** The mirrored temporally-blurred HR model could not account for the data in neither conditions. To compare model fits between early and later parts of the experiment, the models fitted to data from the first two experimental blocks of every condition (acquired on day 1) were fitted to RT from the last two blocks of every condition (acquired on day 2). Since RT on day 2 was faster than on day #1, the models from day 1 were fitted to data from day 2 allowing for one free parameter, the offset, b. The scaling parameter was fixed at $a = 1$ in the linear fitting formula: $f(x) = ax + b$. In each plot the estimated value of b, i.e. a measure of the change in RT between days, is given.

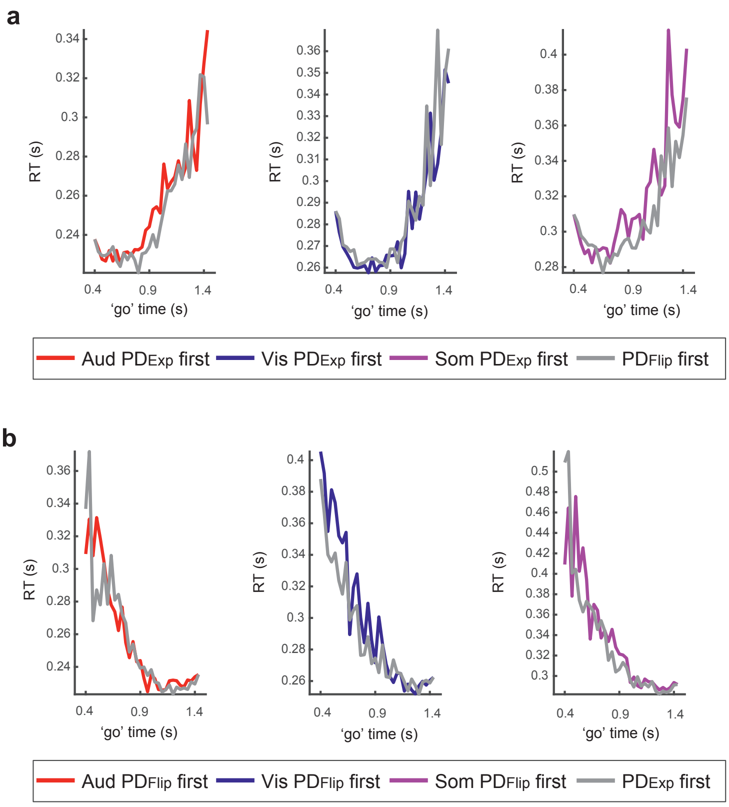

Supplementary Figure 7. Analysis investigating order effects. **a** Comparison between average RT from subjects experiencing the exponential distribution, PD_{Exp}, first (colored curves) and from those experiencing the flipped exponential distribution, PD_{Flip}, first (grey curves). **b** Comparison between average RT from subjects experiencing the flipped exponential distribution, PD_{Flip}, first (colored curves) and from those experiencing the exponential distribution, PD_{Exp}, first (grey curves). No substantial influence of the type of the first experimental condition on the shape of the RT curves was found. To allow for better comparison between the shapes of the two curves in each plot, the curves were aligned with respect to the 'go' time with the highest probability (0.4 s in PD_{Exp} and 1.4 s in PD_{Flip}) by substracting the PD_{Exp} RT curve from the PD_{Flip} RT curve.

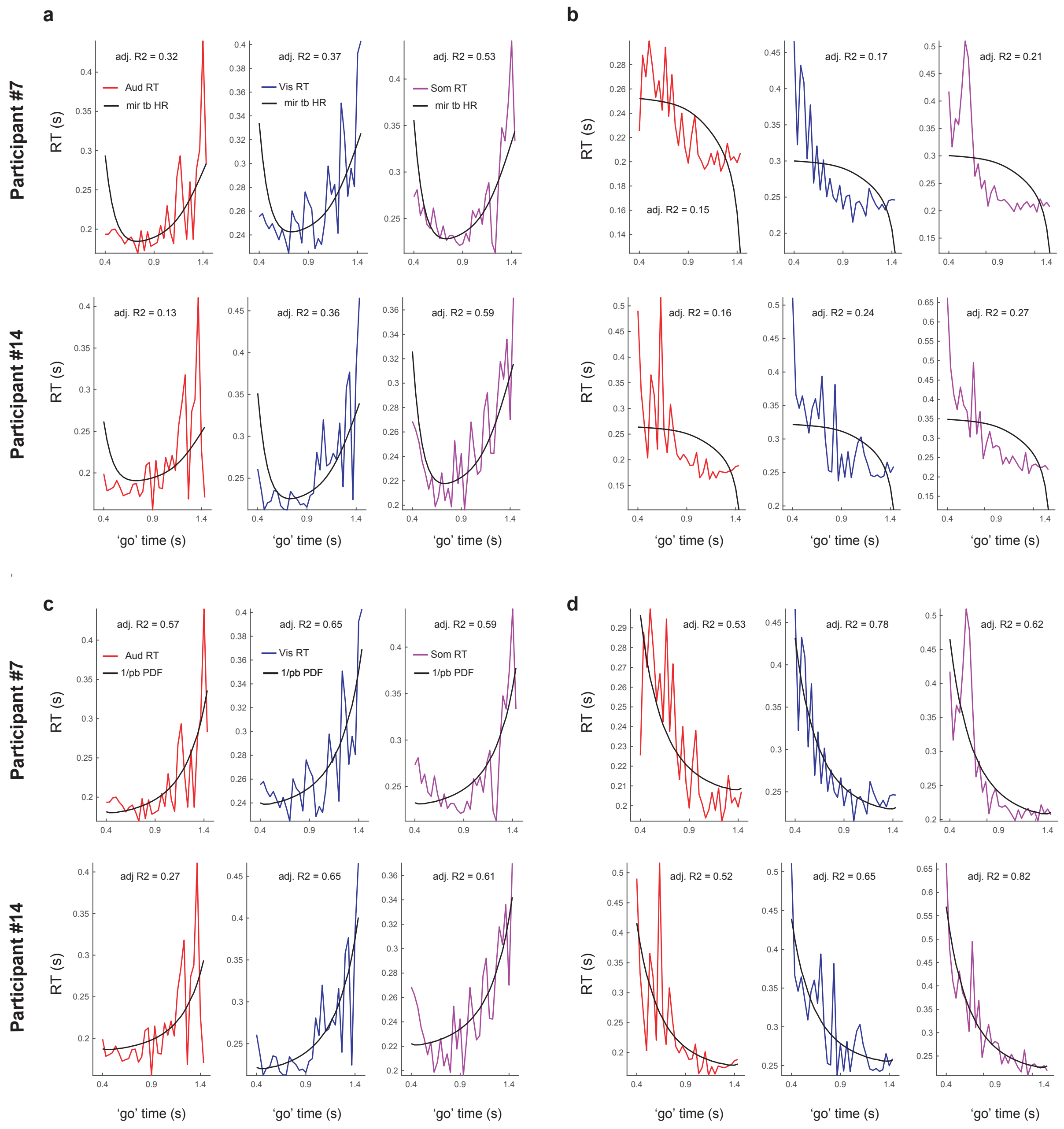

Supplementary Figure 8. Fits of the HR-based and the PDF-based models to single-subject RT data. **a** The mirrored, temporally-blurred HR model captured qualitative aspects of the data in the exponential condition, PD_{Exp}, but it failed to account for the data in the flipped exponential condition, PD_{Flip}, see **b**. **c** In contrast, the reciprocal, probabilistically-blurred PDF model captured data in the PD_{Exp} condition and in the PD_{Flip} condition, see **d.** The behavior of the RT curves of the two participants (#7 and #14) was very similar to the RT curves averaged across all participants Figs. 3 and 4).

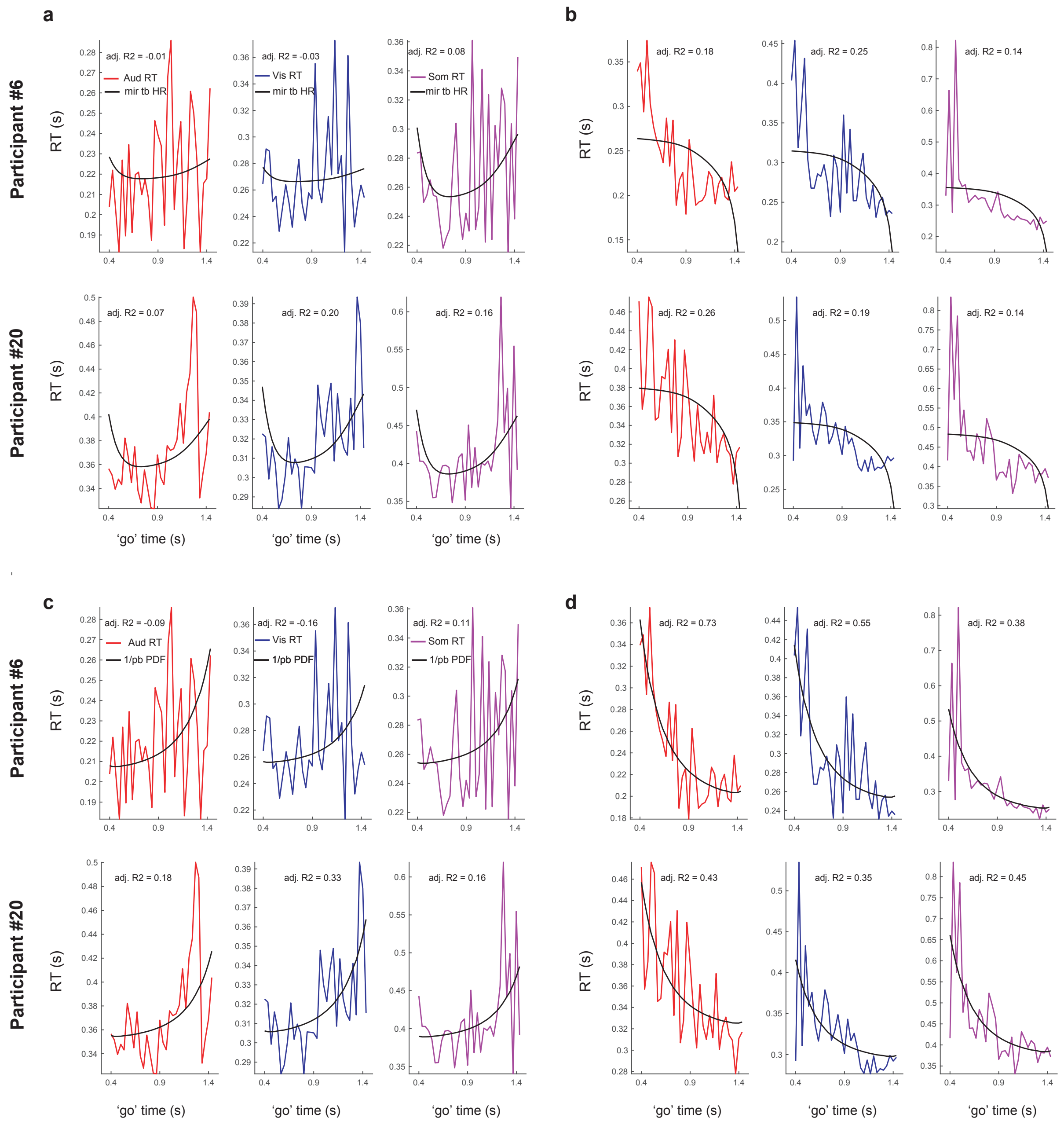

Supplementary Figure 9. Fits of the HR-based and the PDF-based models to single-subject RT data. To demonstrate the variety between different participants' data, RT curves of two participants (#6 and #20) whose data were noisier than those in Supplementary Fig. 8 are shown. Overall, the single-subject RT curves' shapes were very similar to the RT curves averaged across all participants (**Figs. 3 and 4**). **a** The mirrored, temporally-blurred HR model captured qualitative aspects of the data in the PD_{Exp} condition but it failed to account for the data in the PD_{Flip} condition, see **b**. **c** In contrast, and despite the increased noise in the data, the reciprocal, probabilistically-blurred PDF model captured data in PD_{Exp} condition and in the PD_{Flip} condition, see **d**.

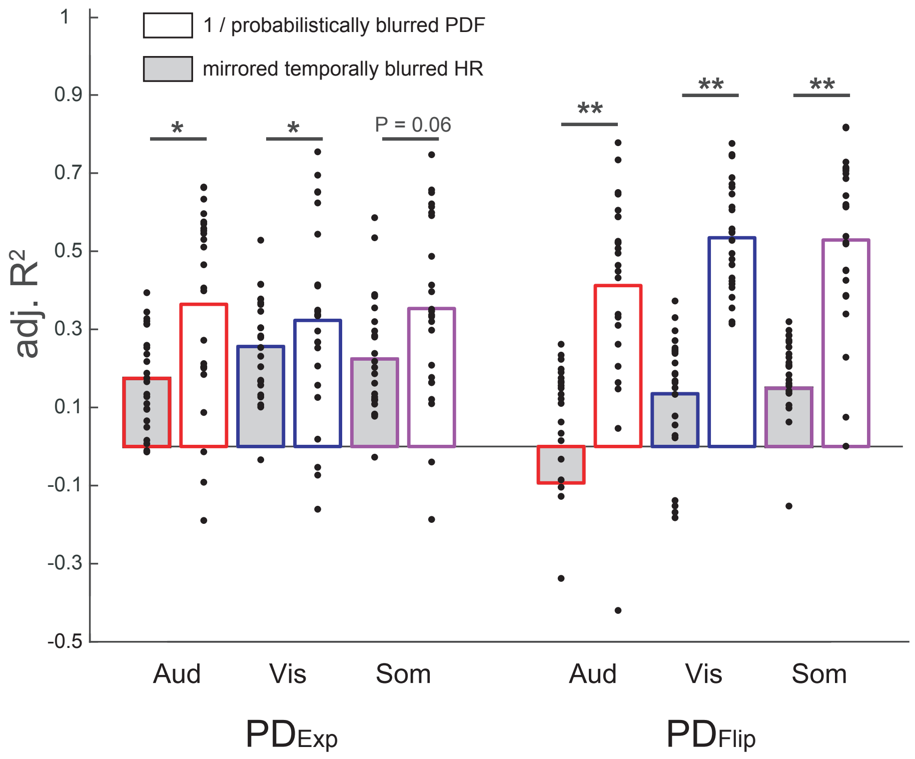

Supplementary Figure 10. Analysis of single-subject goodness-of-fit. Based on single-subject fits, the PDF-based model outperformed the HR-based model in all experimental conditions. Bar plot depicting mean adjusted R^2 from the fits to single-subject data. ** P < 0.001, * P < 0.05; two-tailed Student's *t*-test. Dots represent single-subject values. To allow for plotting over a reasonable range, the following 4 outliers were removed from plots in two conditions: mirrored temporally blurred HR, audition PD_{Flip}: outlier #1 adj. R^2 = - 0.91, outlier #2 adj. R^2 = - 0.93, outlier #3 adj. R^2 = - 2.12, and mirrored temporally blurred HR, somatosensation PD_{Flip}: outlier #4 adj. R^2 = - 0.76.

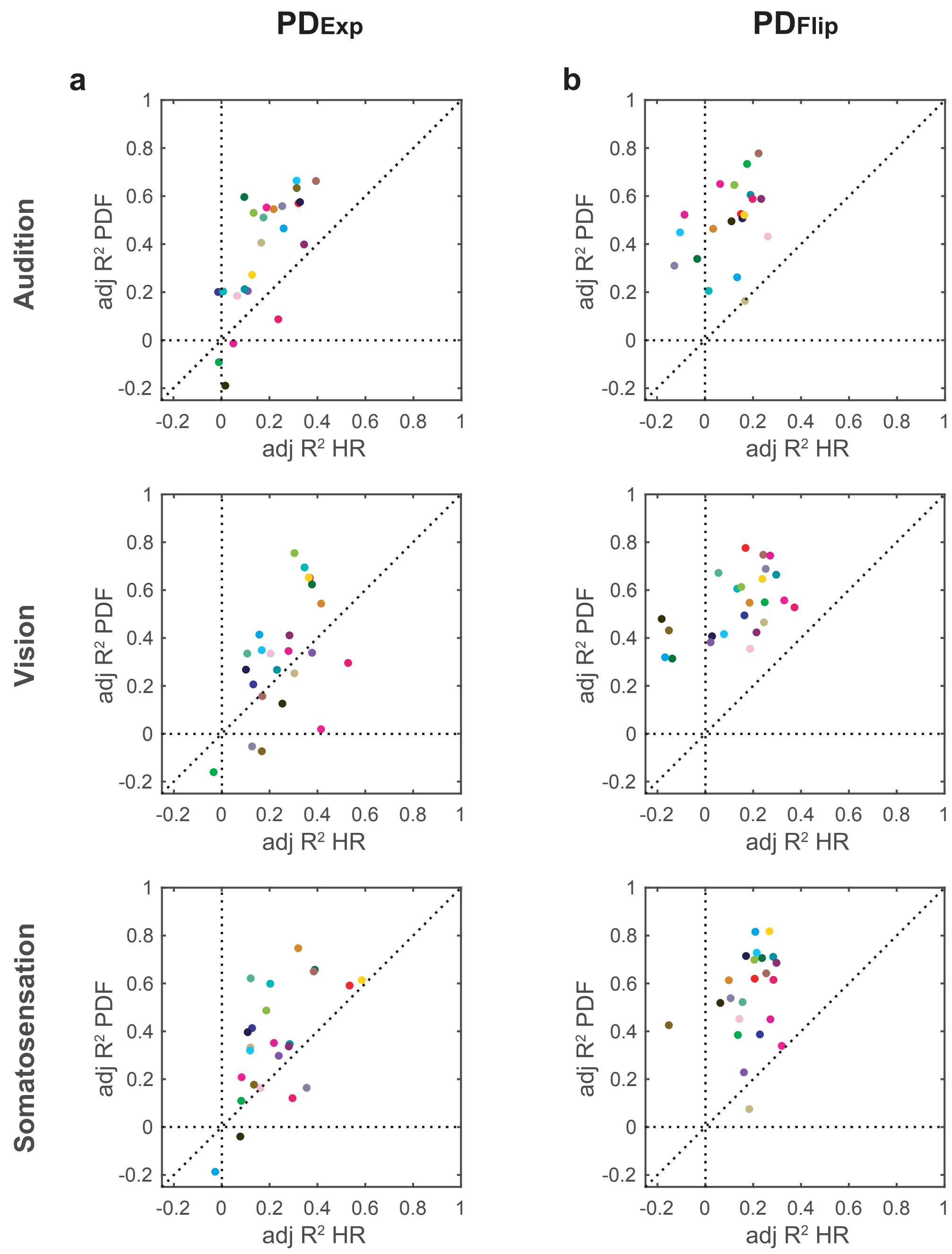

Supplementary Figure 11. Scatter plot of individual participants' adjusted R^2. **a** In the exponential condition, PD_{Exp}, the reciprocal, probabilistically-blurred PDF-based model yielded a highter adjusted R^2 than the the mirrored, temporally-blurred HR-based model in the majority of single-subject fits. **b** As **a** but for the flipped exponential condition, PD_{Flip}.To allow for plotting over a reasonable range, the following 5 drastic outliers were removed from plots in two conditions: Audition PD_{Flip}: outlier #1 adj. R_2 = -2.115 (HR), adj. R_2 = 0.1474 (PDF); outlier #2 adj. R_2 = -0.9267 (HR), adj. R_2 = 0.04645 (PDF); outlier #3 adj. R_2 = -0.9045 (HR), adj. R_2 = - 0.4197 (PDF); outlier #4 adj. R_2 = -0.3377 (HR), adj. R_2 = 0.3311 (PDF). Som Flip: outlier #5 adj. R_2 = -0.7635 (HR), adj. R_2 = 0.0008 (PDF).

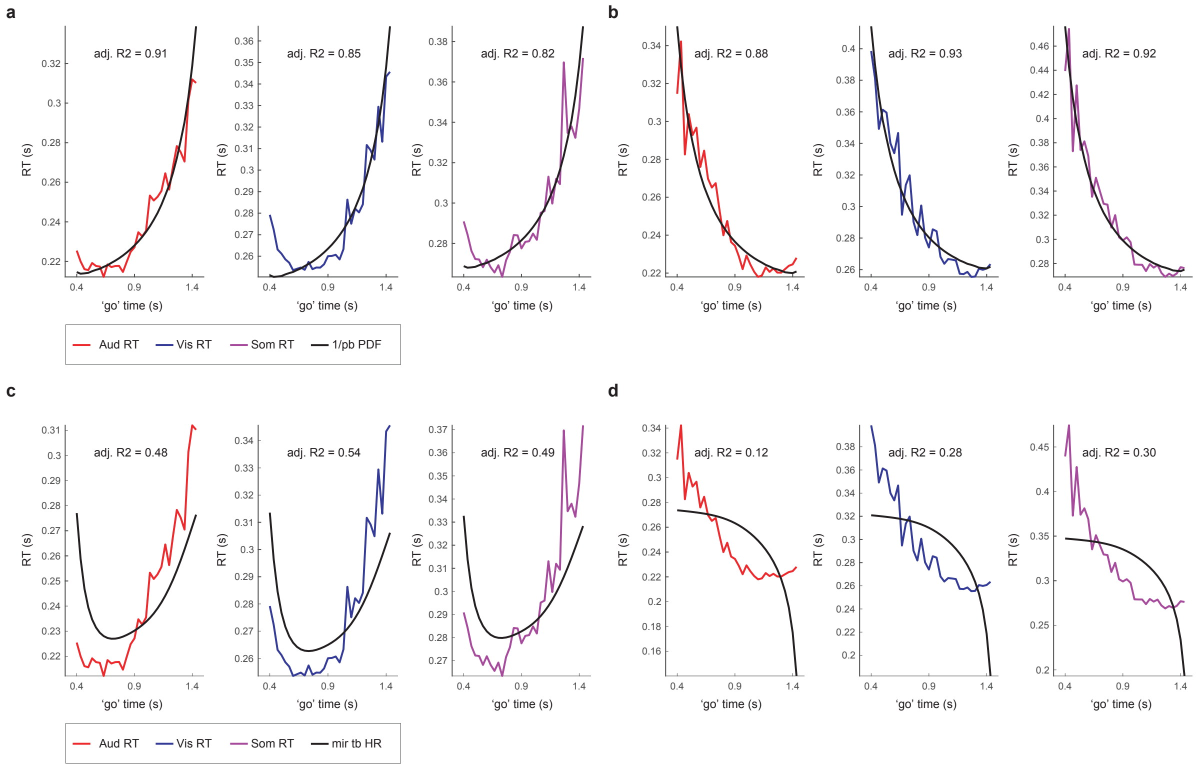

Supplementary Figure 12. Group-level model fits based on individual subjects' Phi for the Gaussian blurring kernel. Within subject, mirrored, temporally-blurred HR-based models and reciprocal, probabilistically-blurred PDF-based models of RT were build for a range of Phi values, [0.1:0.01:0.3]. Adjusted R^2 was calculated for each model over the range of Phi. For each subject the value of Phi that provided the smallest variance in adjusted R^2 across the six experimental conditions was selected as the subject-specific Phi. Using this subject-specific value of Phi, both HR-based and PDF-based models were constructed again in each of the six experimental conditions within each subject. Across subjects, the fitted subject-specific models were averaged for each 'go' time and plotted against group-level RT. Adjusted R^2 of the resulting group-level model was calculated. PDF-based model in the exponential condition, see **a**, and in the flipped exponential condition, see **b**, outperformed the HR-based model, see **c** and **d**. Importantly, the construction of the models based on subject-specific values for Phi yielded very similar results compared to the models based on a fixed value of Phi = 0.21 for group-level fits (Fig. 3 a and b and Fig 4c and d). The average subject-specific value was $\varphi = 0.2054 \pm 0.0622$ (mean ± standard deviation, SD) for the mirrored, temporally-blurred HR model and the corresponding value was $\varphi = 0.1846 \pm 0.0798$ (mean ± SD) for the reciprocal, probabilistically-blurred PDF. Taken together, these findings indicate that the Gaussian kernel based on Phi = 0.21, as was used on the group-level fits in Figs. 3 and 4, gave a good approximation of the timing behavior averaged across subjects.

Supplementary Note 2

Control experiment without catch trials. To investigate event anticipation in a setting without catch trials, where every trial contains a 'go' cue, a control experiment was conducted. Using the same 'set'- 'go' task as described above, auditory and visual blocks of trials were presented. Half of the blocks followed the exponential distribution (PD_{Exp}), the other half followed its flipped counterpart (PD_{Flip}). No catch trials were presented. To minimize sequential effects, the 'go' times were randomized with the constraint that no more than two consecutive trials had the same 'go' time. The intertrial interval (ITI, range 1.4 to 2.4 s) was randomly drawn from a uniform distribution. The probability distribution changed after two blocks without notification. To control for order effects, the conditions (sensory modalities and probability distributions) were organized in a Latin square design, based on which modality and distribution were shuffled across subjects. A new group of 12 subjects was invited and performed the task. Per sensory modality, each subject produced 240 reaction times under each probabilistic condition, which sum up to a total of 920 reaction times per subject for two modalities and two probabilistic conditions.

In the experiment without catch trials, an event will have occurred by the end of a timespan and accordingly, the uncertainty of event occurrence is zero. Note that in both contexts with and without catch trials there is still uncertainty about *when* the event occurs. The HR is commonly endorsed for both contexts without[2-6] and with catch trials[7,8] as a model of RT. Clearly, the "temporally-blurred, mirrored HR" did not fit the data (Supplementary Fig. 13a and b), whereas the "probabilistically-blurred, reciprocal PDF" captured the data adequately in all conditions (Supplementary Fig. 13c and d). We thus conclude that in both contexts, with (Fig. 3 and 4) and without catch trials, the PDF-based model outperformed the HR-based model.

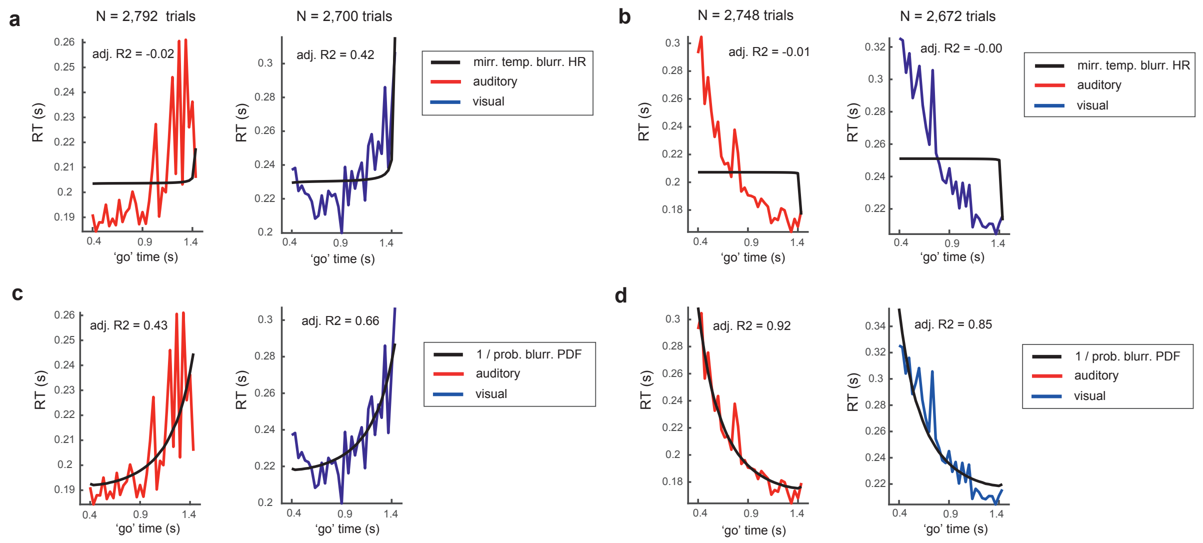

Supplementary Figure 13. Control experiment without catch trials. **a** In setting without catch trials, models based on mirrored, temporally-blurred hazard rate fail to capture RT data in the exponential condition, PD_{Exp}. **b** As **a** but for flipped exponential condition, PD_{Flip}. **c** In contrast, models based on the reciprocal, probabilistically-blurrd PDF fit the data well in the PD_{Exp} condition. **d** As **c** but for PD_{Flip}. Auditory (red) and visual (blue) reaction time data from 12 participants who performed the 'set' - 'go' task without catch trials. In this setting, where there in no uncertainty about 'go' cue occurrence, PD_{Exp} and PD_{Flip} distributions of 'go' time were presented in audition and in vision.

Supplementary Note 3

Gaussian control experiment. 18 of the original 24 subjects were reinvited and participated in a third experimental session with a Gaussian distribution of 'go' times. The subjects performed auditory, visual, and somatosensory 'set' - 'go' trials, which included 9.09 % catch trials. The time between 'set' and 'go' was drawn from a Gaussian distribution with parameters $\mu = 0.9$ and $\sigma = 0.25$. The distribution was truncated at the flanks, giving a range of 'go' times from 0.4 to 1.4 s, giving the distribution a spread of two standard deviations around the mean. To minimize sequential effects, the 'go' times were randomized with the constraint that no more than two consecutive trials had the same 'go' time. The intertrial interval (ITI, range 1.4 to 2.4 s) was randomly drawn from a uniform distribution. To control for order effects, the order of sensory conditions was shuffled across subjects. Each subject generated 300 reaction times per sensory modality.

Again, the temporally-blurred, mirrored HR could not adequately account for the data (Supplementary Fig. 14a). In contrast, the probabilistically-blurred, reciprocal PDF captured the behavior of the data qualitatively in all three modalities (Supplementary Fig. 14b). As the figure shows, the fit was best in the auditory condition, similar to the exponential and flipped exponential distributions. Although the fits to visual and somatosensory data were less accurate, the PDF-based model captured the qualitative features of the biphasic RT modulation. Interestingly, in these two modalities the deviations between data and model seemed to be dominated by an upward bend in RT at short 'go' times. This pattern resembled the deviations between data and model observed in the exponential condition, and most importantly, only in vision and somatosensation (Fig. 4c). In the case of the flipped exponential distribution (Fig. 4d), the bend in the RT curve at shorter 'go' times is not observable directly because the longest RTs are at the shortest 'go' times. Closer inspection reveals that the RTs in vision and somatosensation at the shorter 'go' times are much longer than in audition. These findings hint at potential modality-specific effects at shorter 'go' times, which are manifested more in vision and somatosensation than audition.

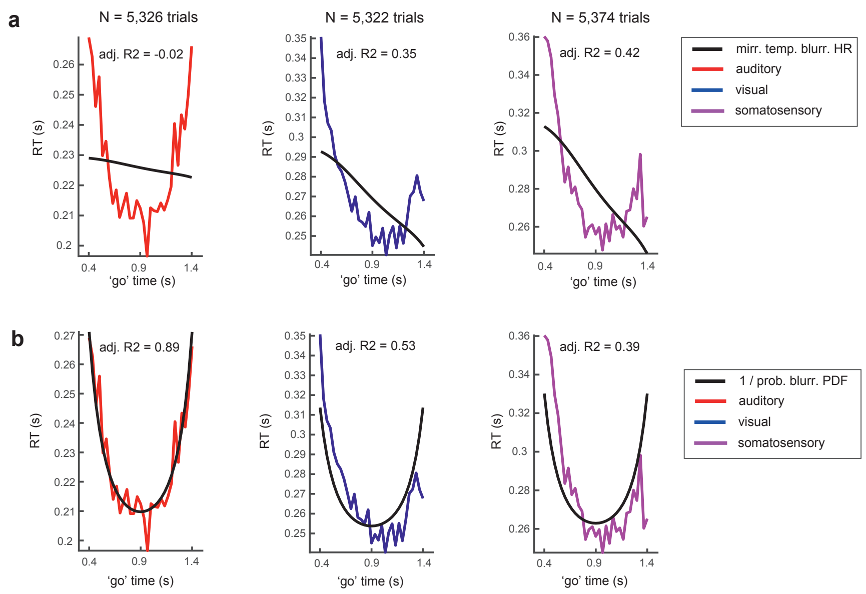

Supplementary Figure 14 | In a Gaussian condition, PDF-based models outperform HR-based models. Auditory (red), visual (blue), and somatosensory (violet) RT data from 18 participants performing the task with a Gaussian distribution of 'go' times (see Methods). **a** Fits of the *temporally-blurred, mirrored hazard rate* to RT. **b** Fits of the *probabilistically-blurred, reciprocal PDF* to RT.

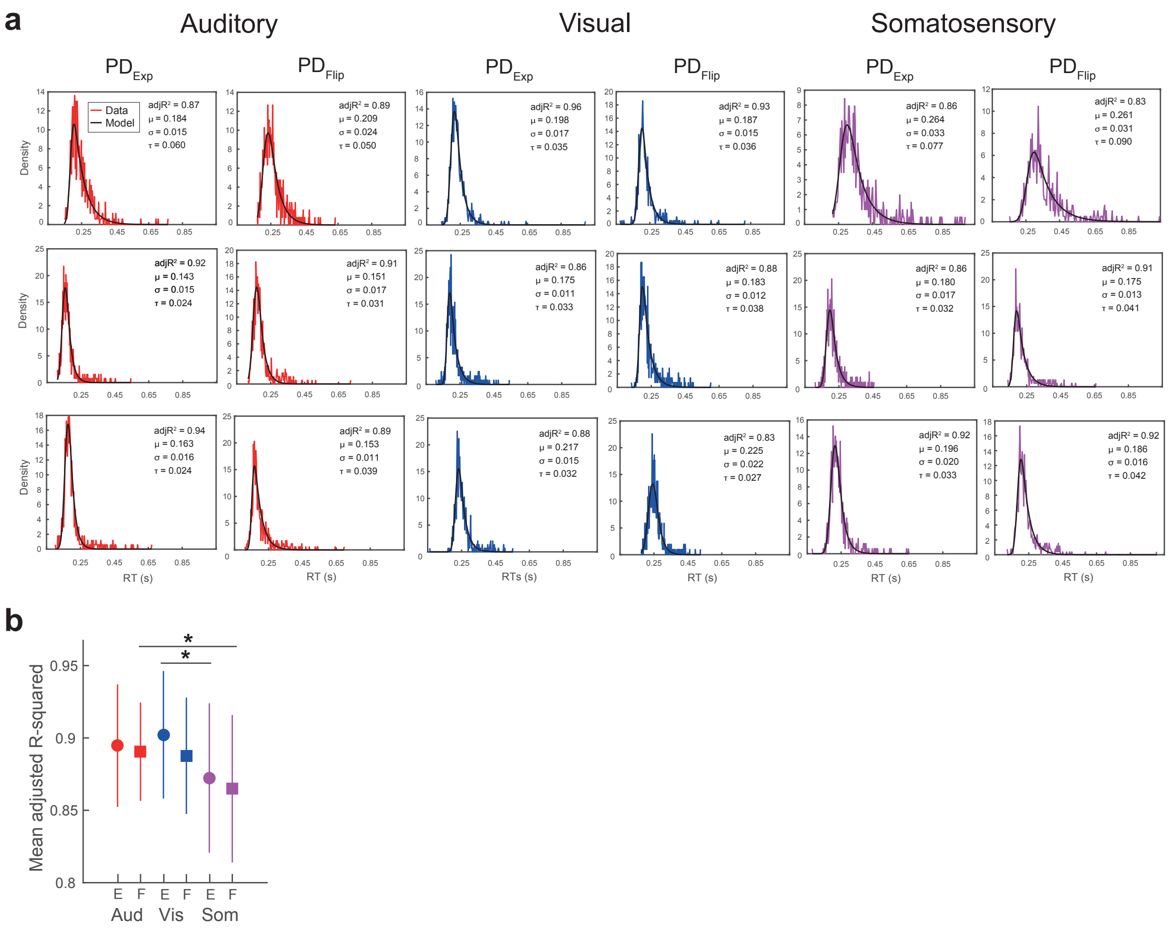

Supplementary Figure 15. Single-subject fits of exponential-Gaussian model to RT distributions. **a** Each row contains data for a single subject. In the three sensory modalities (red = audition, blue = vision, violet = somatosensation) and in the two experimental conditions (PD_{Exp} and PD_{Flip}), the ex-Gaussian model captured the RT distributions. **b** Goodness-of-fit assessed at the group level averaging adjusted R^2 from the individual single-subject fits indicates that the ex-Gaussian model captured the data well. (planned contrasts, * $P < 0.05$, two-tailed Student's t test) Error bars denote standard deviation.

Supplementary Table 1. Three-way analysis of variance (ANOVA) on factors probability distribution, sensory modality and day.

	Probability distribution		Sensory modality		Day	
	$F_{(1,287)}$	P	$F_{(2,287)}$	P	$F_{(1,287)}$	P
median RT	0.25	0.64	41.70	$1.3*10^{-16}$	22.10	$4.1*10^{-6}$
IQR_{RT}	8.40	0.0041	14.50	$1.0*10^{-6}$	29.30	$1.3*10^{-7}$
μ	0.42	0.52	63.8	$1.3*10^{-23}$	11.80	0.0007
σ	0.93	0.34	12.40	$7.1*10^{-6}$	3.99	0.049
τ	6.51	0.011	10.82	$3.0*10^{-5}$	30.64	$7.1*10^{-8}$

A separate three-way ANOVA was performed for median RT, interquartile range of RT (IQT_{RT}), and for estimates of exponential parameter, τ, and Gaussian parameters, μ and σ, from the exponential-Gaussian model fits. All three-way and two-way interactions were n.s. and were therefore removed from all above models.

Supplementary Table 2. *t* tests on significant factor *probability distibution* (three-way ANOVA, planned contrasts).

	Auditory	$t_{(23)}$	*P*	Visual	$t_{(23)}$	*P*	Somato.	$t_{(23)}$	*P*
Δ IQR_{RT}	-10.3 (19.4)	-2.62	0.0154	-11.7 (20.2)	-2.85	0.0091	-11.4 (12.7)	-4.39	0.00022
$\Delta\ \tau$	-9.7 (18.5)	-2.58	0.0166	-9.5 (17.5)	-2.67	0.0136	-8.2 (13.7)	-2.92	0.0077

Differences in variables between exponential and flipped exponential conditions (PD_{Exp} - PD_{Flip}). All variables are in units of ms, all variances (in parentheses) are standard deviations.

Supplementary Table 3. *t* tests on significant factor *sensory modality* within exponential condition (three-way ANOVA, planned contrasts).

	Aud. - Vis.	$t_{(23)}$	*P*	Aud. - Som.	$t_{(23)}$	*P*	Vis. - Som.	$t_{(23)}$	*P*
Δ median RT	-43.2	-8.7	$1.1 * 10^{-8}$	-55.0	-9.2	$3.4 * 10^{-9}$	-11.7	-1.6	0.12
	(24.5)			(29.2)			(35.6)		
Δ IQR_{RT}	7.5	1.78	0.089	-14.1	-3.56	0.0019	-21.5	5.80	$6.6*10^{-6}$
	(20.5)			(19.4)			(18.2)		
Δ μ	-47.7	-11.6	$4.1*10^{-11}$	-44.9	-8.9	$6.4*10^{-9}$	2.8	0.49	0.64
	(20.1)			(24.7)			(28.8)		
Δ σ	1.3	-0.98	0.34	-6.7	-3.17	0.0043	-5.3	-2.66	0.014
	(6.7)			(10.3)			(9.8)		
Δ τ	7.1	1.58	0.13	-12.5	-3.01	0.006	-19.5	-6.01	$3.9 *10^{-6}$
	(22.0)			(20.3)			(15.9)		

Differences in variables across sensory modalities within $PD_{Exp.}$ All variables are in units of ms, all variances (in parentheses) are standard deviations.

Supplementary Table 4. *t* tests on significant factor *sensory modality* within flipped exponential condition (three-way ANOVA, planned contrasts).

	Aud. - Vis.	$t_{(23)}$	P	Aud. - Som.	$t_{(23)}$	P	Vis. - Som.	$t_{(23)}$	P
Δ median RT	-41.7	-7.0	$3.7 * 10^{-7}$	-54.7	-7.0	$4.1 * 10^{-7}$	-13.1	-1.3	0.20
	(29.0)			(38.4)			(47.9)		
Δ IQR_{RT}	6.0	0.84	0.41	-15.1	-2.31	0.0302	-21.2	-3.14	0.0047
	(35.5)			(32.0)			(33.1)		
Δ μ	-45.9	-10.5	$2.8*10^{-10}$	-43.9	-7.4	$1.7*10^{-7}$	2.1	0.29	0.78
	(21.3)			(29.1)			(34.8)		
Δ σ	-1.1	-0.82	0.42	-9.3	-3.87	0.0008	-8.2	-3.42	0.0024
	(6.7)			(11.8)			(11.8)		
Δ τ	7.3	1.07	0.30	-10.9	-1.72	0.10	-18.2	- 2.98	0.0068
	(33.3)			(31.2)			(30.0)		

Differences in variables across sensory modalities within $PD_{Flip.}$ All variables are in units of ms, all variances (in parentheses) are standard deviations.

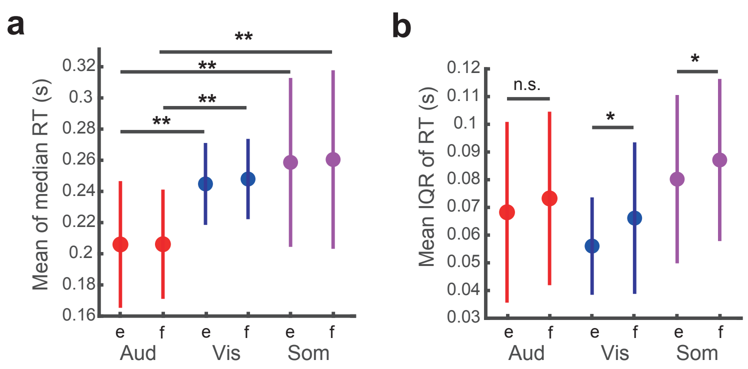

Supplementary Figure 16. Analysis of RT data excluding 'go' times featuring an upward bend in RT curves (**Fig**. 4c). RT from 'go' times smaller than 0.5667 s were discarded from all conditions to remove the effect of the observed upward bend in RT at the short 'go' time range of 0.4 to 0.535 s. **a** The mean of the median RT significantly differed between audition and vision and between audition and somatosensation in both exponential ("e") and flipped exponential ("f") conditions. Within sensory modality, the mean of the median RT did not differ between the two conditions (audition: $P = 0.96$, vision: $P = 0.13$, somatosensation: $P = 0.61$; two-tailed Student's t test). **b** Mean interquartile range (IQR) of RT differed across exponential and flipped exponential distributions in vision and somatosensation. In audition, the mean of the IRT of RT was larger in the flipped exponential than in the exponential distribution, but the effect did not reach significance ($P = 0.27$; two-tailed Student's t test). This lack of a difference could not have been caused by an upwards bend in the RT curve because such a prominent bend does not exist in the auditory condition. Instead, it is probably caused by the truncation which removed a lot of data points. Taken together, these above analyses indicate that the upward bend in RT at shorter 'go' times does not impact the previous results derived from the full dataset (**Fig. 7b and c**). Error bars denote standard deviation.

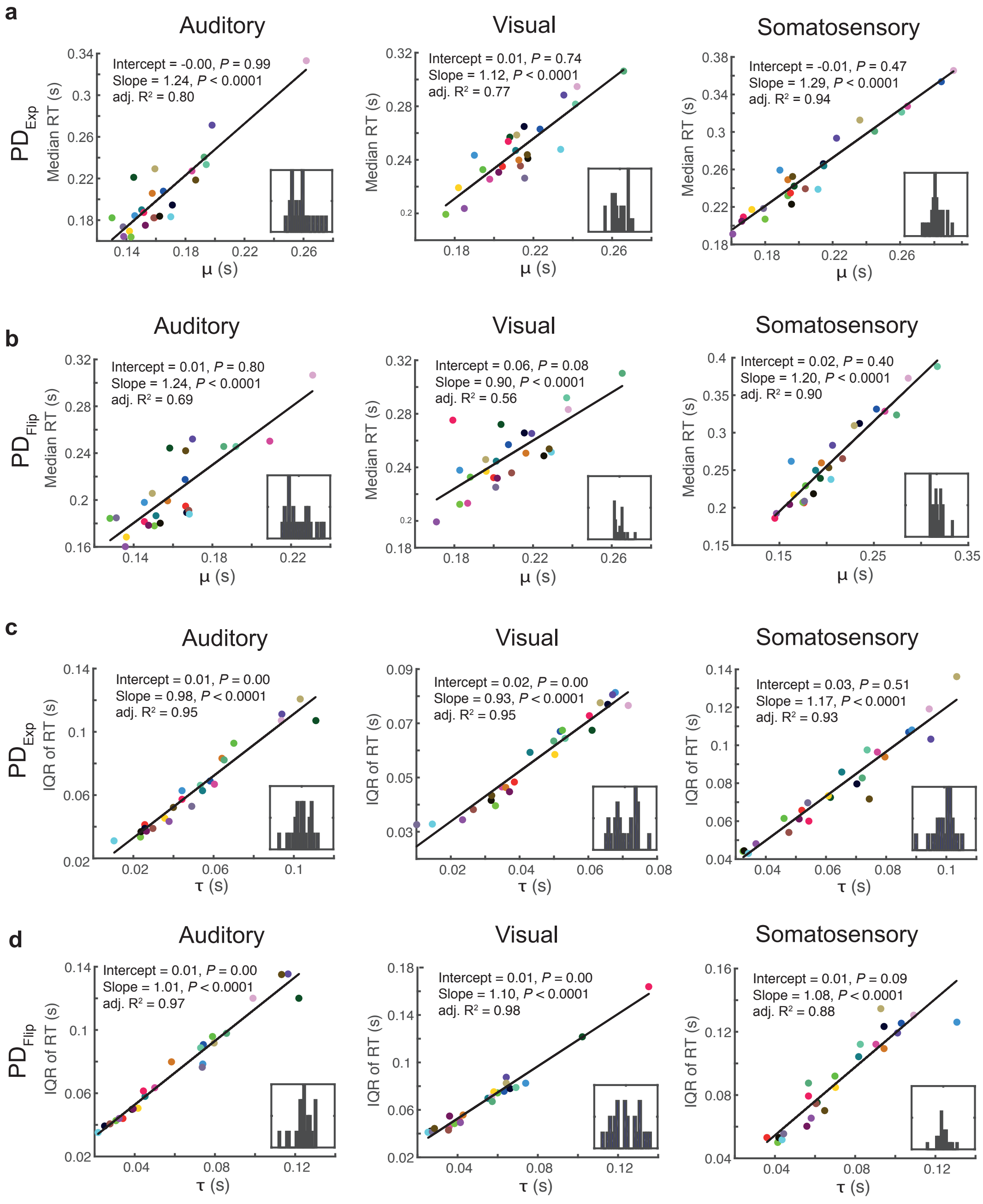

Supplementary Figure 17. Exponential-Gaussian fit parameters μ and τ capture average RT and variance of RT. **a** In the PD_{Exp} condition, the relationship between Gaussian μ and median RT is captured adequately by a linear regression model (black fit line). **b** As **a** but for PD_{Flip} **c** Similarly, in the PD_{Exp} condition, the relationship between exponential τ and IQR_{RT} is captured by a linear regression model (black fit line). **d** As **c** but for PD_{Flip}. Each dot represents a single subject. Inset graphs show residuals of linear fit. All P values: t test in linear regression.

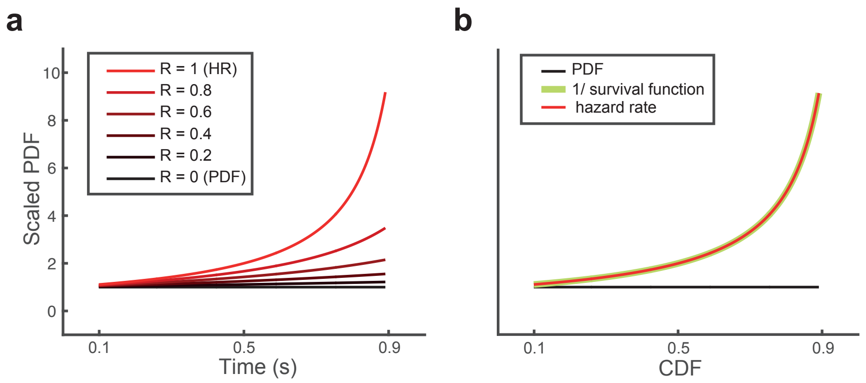

Supplementary Figure 18. Hypothesized effect of reward expectation on probability density and influence of survival function on hazard rate. **a** Influence of R, a factor hypothesized to reflect reward expectancy, on probability density. The parameter PDF_s described by the formula $PDF_s = \frac{PDF}{(1 - R \times CDF)}$, is termed here the *scaled PDF* in which the term $\frac{1}{(1 - R \times CDF)}$ is the scaling factor. In the case of no expected reward, R approaches 0, the scaling factor approaches a constant value across time, and the *scaled PDF* approaches the PDF itself. In the case of high reward expectation, R approaches 1 and the *scaled PDF* approaches the HR, as $HR = \frac{PDF}{(1 - CDF)}$. **b** Functions of a uniform PDF that spans from zero to one. In the formula for the HR, $HR = \frac{1}{surival\ function} \times PDF$, the scaling term $\frac{1}{surival\ function}$ monotonically increases, with the highest gradient occuring when CDF approaches one at the right extremum of any distribution. These high scaling values drive the hazard rate to also sharply increase (red line). This highlights the dominant role of the term $\frac{1}{surival\ function}$ on the values of HR towards the right extremum of PDFs. Especially in the absence of catch trials, where the CDF approaches 1 asymptotically, the factor $\frac{1}{surival\ function}$ takes on extremely high values near the end of the distribution ($survival\ function = 1 - CDF$), thus outweighting the impact of the PDF (at least for the majority of PDFs). For plotting, the functions have been truncated to avoid extreme values at the right extremum. The y-axis accomodates probability density, cumulative probability density and hazard rate, therefore no units are specified.

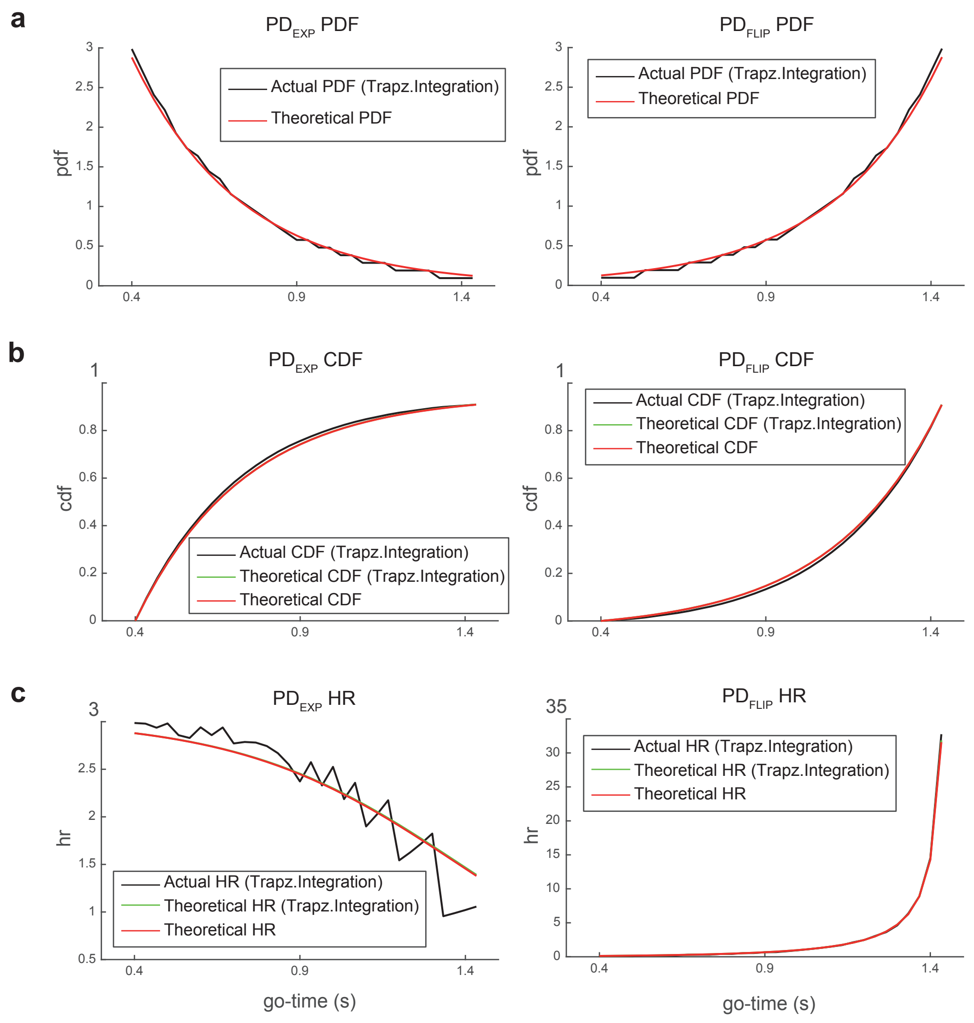

Supplementary Figure 19. Comparison of theoretical PDF, CDF and hazard rate (HR) with the discretized versions presented in the experiment. The discretized versions of the CDF were derived by trapezoidal integration of the discretized PDF. **a** The discretized versions of the PDF approximated closely the theoretical ones (notice the step-like shape). **b** The same was true for the CDF (since the discretized CDF is the integral of the discretized PDF it is not itself a step function but rather a piecewise linear function). **c** The jigsaw-pattern in the HR of the PD_{Exp} condition is a consequence of the discretization of the PDF while the CDF remains continuous. Regardless of these jigsaw-pattern the discretized HR curve follows the theoretical one.

Supplementary Citations

1 Mauk, M. D. & Buonomano, D. V. The neural basis of temporal processing. *Annu Rev Neurosci* **27**, 307-340, (2004).

2 Janssen, P. & Shadlen, M. N. A representation of the hazard rate of elapsed time in macaque area LIP. *Nat Neurosci* **8**, 234-241, (2005).

3 Cravo, A. M., Rohenkohl, G., Wyart, V. & Nobre, A. C. Endogenous modulation of low frequency oscillations by temporal expectations. *J Neurophysiol* **106**, 2964-2972, (2011).

4 Cui, X., Stetson, C., Montague, P. R. & Eagleman, D. M. Ready...go: Amplitude of the FMRI signal encodes expectation of cue arrival time. *PLoS Biol* **7**, e1000167, (2009).

5 Sharma, J. *et al.* Spatial Attention and Temporal Expectation Under Timed Uncertainty Predictably Modulate Neuronal Responses in Monkey V1. *Cereb Cortex* **25**, 2894-2906, (2015).

6 Fiorillo, C. D., Newsome, W. T. & Schultz, W. The temporal precision of reward prediction in dopamine neurons. *Nat Neurosci* **11**, 966-973, (2008).

7 Ghose, G. M. & Maunsell, J. H. Attentional modulation in visual cortex depends on task timing. *Nature* **419**, 616-620, (2002).

8 Schoffelen, J. M., Oostenveld, R. & Fries, P. Neuronal coherence as a mechanism of effective corticospinal interaction. *Science* **308**, 111-113, (2005).
