# Supplementary material for: The anticipation of events in time
Source: Nat Commun. 2019 Dec 20;10:5802. doi: 10.1038/s41467-019-13849-0 (PMC6925136; doi:10.1038/s41467-019-13849-0)
Supplement: Supplementary file 3 — Reporting Summary [file 41467_2019_13849_MOESM3_ESM.pdf]

nature research

Corresponding author(s): David Poeppel

Last updated by author(s): 2019 11 11

Reporting Summary

Nature Research wishes to improve the reproducibility of the work that we publish. This form provides structure for consistency and transparency in reporting. For further information on Nature Research policies, see Authors & Referees and the Editorial Policy Checklist .

Statistics

For all statistical analyses, confirm that the following items are present in the figure legend, table legend, main text, or Methods section.

n/a	Confirmed	
☐	☒	The exact sample size (*n*) for each experimental group/condition, given as a discrete number and unit of measurement
☐	☒	A statement on whether measurements were taken from distinct samples or whether the same sample was measured repeatedly
☐	☒	The statistical test(s) used AND whether they are one- or two-sided<br>*Only common tests should be described solely by name; describe more complex techniques in the Methods section.*
☒	☐	A description of all covariates tested
☐	☒	A description of any assumptions or corrections, such as tests of normality and adjustment for multiple comparisons
☐	☒	A full description of the statistical parameters including central tendency (e.g. means) or other basic estimates (e.g. regression coefficient) AND variation (e.g. standard deviation) or associated estimates of uncertainty (e.g. confidence intervals)
☐	☒	For null hypothesis testing, the test statistic (e.g. *F*, *t*, *r*) with confidence intervals, effect sizes, degrees of freedom and *P* value noted<br>*Give P values as exact values whenever suitable.*
☒	☐	For Bayesian analysis, information on the choice of priors and Markov chain Monte Carlo settings
☒	☐	For hierarchical and complex designs, identification of the appropriate level for tests and full reporting of outcomes
☒	☐	Estimates of effect sizes (e.g. Cohen's *d*, Pearson's *r*), indicating how they were calculated

Our web collection on statistics for biologists contains articles on many of the points above.

Software and code

Policy information about availability of computer code

Data collection	Data was collected using Psychophysics Toolbox Version 3, http://psychtoolbox.org/ running under MatLab 2014b (The MathWorks, Natick MA, USA)
Data analysis	Data analysis was exclusively performed in MatLab (The MathWorks, Natick MA, USA), using mostly version 2018b and the commercially available toolboxes (e.g. Statistics Toolbox)

For manuscripts utilizing custom algorithms or software that are central to the research but not yet described in published literature, software must be made available to editors/reviewers. We strongly encourage code deposition in a community repository (e.g. GitHub). See the Nature Research guidelines for submitting code & software for further information.

Data

Policy information about availability of data

All manuscripts must include a data availability statement. This statement should provide the following information, where applicable:

- Accession codes, unique identifiers, or web links for publicly available datasets
- A list of figures that have associated raw data
- A description of any restrictions on data availability

The data that support the findings of this study are available from the corresponding author upon reasonable request.

Field-specific reporting

Please select the one below that is the best fit for your research. If you are not sure, read the appropriate sections before making your selection.

☒ Life sciences ☐ Behavioural & social sciences ☐ Ecological, evolutionary & environmental sciences

Life sciences study design

All studies must disclose on these points even when the disclosure is negative.

Sample size

The study aimed to quantify on the group-level effects of temporal anticipation. The sample size (24 participants) was determined based on the randomization procedure described below.

Data exclusions

4 participants did not finish the 2-day experiment due to inability to restrict blinking to the intertrial interval (N=2), malfunctioning of experimental set-up (N=1), and tiredness (N=1). These 4 participants were removed from the analysis and 4 different participants were tested to complete the group of 24.
The experiment used a simple reaction time (RT) task. These tasks are known to produce short RTs (Luce, 1986). Therefore RTs outside of the range of 0.05 to 1.05 s were removed from the analysis. Experimental trials during which visual fixation requirements were violated (see Methods) were discarded from the analysis.

Replication

The key findings of the paper (e.g. Fig. 4c and d) were replicated in a different group of 24 subjects with a sligthly altered design (fewer trials, 'go' times presented at a temporal resolution of 1/60 s etc.).

Randomization

To control for order effects, the conditions (sensory modalities and probability distributions) were organized in a Latin square design, based on which modality and distribution were shuffled and balanced across subjects and days.

Blinding

No blinding of the investigators during data acquisition and analysis was used.

Reporting for specific materials, systems and methods

We require information from authors about some types of materials, experimental systems and methods used in many studies. Here, indicate whether each material, system or method listed is relevant to your study. If you are not sure if a list item applies to your research, read the appropriate section before selecting a response.

Materials & experimental systems

n/a	Involved in the study	
☒	☐	Antibodies
☒	☐	Eukaryotic cell lines
☒	☐	Palaeontology
☒	☐	Animals and other organisms
☐	☒	Human research participants
☒	☐	Clinical data

Methods

n/a	Involved in the study	
☒	☐	ChIP-seq
☒	☐	Flow cytometry
☒	☐	MRI-based neuroimaging

Human research participants

Policy information about studies involving human research participants

Population characteristics

24 human participants (13 female), aged 19-33, participated in the auditory, visual, and somatosensory experiments. 18 subjects (13 female), aged 19-33 participated in an auditory and visual control experiment ("no-catch-trials experiment"). All were right-handed and had normal or corrected-to-normal vision and reported no hearing impairment and no history of neurological disorder.

Recruitment

Participants were recruited using a custom experiment-management software. The software was set to generate a gender-matched group of right-handed subjects whose age ranged between 18 and 35 years out of a pool of several thousand subjects.

Ethics oversight

The experiments were approved by the Ethics Council of the Max-Planck Society

Note that full information on the approval of the study protocol must also be provided in the manuscript.
